# Supplementary material for: The Antitumour Mechanisms of Carotenoids: A Comprehensive Review
Source: Antioxidants (Basel). 2024 Aug 30;13(9):1060. doi: 10.3390/antiox13091060 (PMC11428676; doi:10.3390/antiox13091060)
Supplement: Supplementary file 1 [file antioxidants-13-01060-s001.zip › antioxidants-3160699-supplementary.pdf]

Table S1. Preclinical studies of antiproliferative effects of carotenoids on cancer. This table summarizes the observed effects on cell proliferation, the specific carotenoids studied, the cancer types targeted and the experimental models used.

Carotenoid	Cancer	Model	Effect	Reference
β-carotene	Breast	MDMA-MB-231 cell line	Cell cycle arrest in S phase via JNK	Antunes *et al.*, 2022
	Leukaemia	K562 cell line	Cell cycle arrest in G0/G1 phase via PPARγ and Keap1-Nrf2/EpRE/ARE	Zhang *et al.*, 2011
		HL-60 cell line	Cell cycle arrest in G0/G1 phase by increasing p21 expression	Palozza, 2005
	Colon	COLO 320 HSR, LS-174, HT-29 and WiDr cell lines	Cell cycle arrest in G2/M phase by reduction of cyclin A expression	Palozza *et al.*, 2002
		Xenograft mice injected with HT-116 cells	Suppression of tumour volume and delayed tumour formation	Lee *et al.*, 2022
	Pituitary gland	AtT20 cell line	Cell cycle arrest in G2/M phase by decreasing Skp2 expression and increasing $p27^{kip1}$ expression	Haddad *et al.*, 2013
	Mesothelioma	SPC212 cell line	Reduction of proliferation	Kacar *et al.*, 2022
Lycopene	Breast	MCF-7 and MDA-MB-235 cell lines	Cell cycle arrest in G0/G1 phase	Gloria *et al.*, 2014
		MDA-MB-231 and BT474 cell lines	Cell cycle arrest in G0/G1 phase by decreasing Skp2 expression	Huang *et al.*, 2011
		MCF-7 cell line	Cell cycle arrest in G0/G1 phase by inactivating IGF-I	Karas *et al.*, 2000
		MCF-7 and T-47D cell lines	Cell cycle arrest in G0/G1 phase by decreasing cyclins D1 and D3 expression	Nahum *et al.*, 2001
		MDA-MB-231 cell line	Reduction of proliferation by inhibition of IκB kinase	Assar *et al.*, 2016
		MCF-7 cell line	Cell cycle arrest in G0/G1 phase	Teodoro *et al.*, 2012
	Endometrium	ECC-1 cell line	Cell cycle arrest in G0/G1 phase by decreasing cyclins D1 and D3 expression	Nahum *et al.*, 2001
	Prostate	PC3 cell line	Reduction of proliferation by inhibition of IκB kinase	Assar *et al.*, 2016
		DU145 cell line	Cell cycle arrest in M/G2 phase	Teodoro *et al.*, 2012
	Colon	HT-29 and T84 cell lines	Cell cycle arrest in G0/G1 phase	Teodoro *et al.*, 2012
		Mice xenograft model	Inhibition of growth and progression of colon cancer cells mediated by p21	Tang *et al.*, 2011
	Leukaemia	HL-60 cell line	Cell cycle arrest in G0/G1 phase	Amir *et al.*, 1999
	Stomach	AGS, SGC-7901 and GES-1***** cell lines	Cell growth suppression only in cancer cell lines (AGS, G0/G1 arrest; SGC-7901, M/G2 arrest) by reducing amplification of CCNE1	Zhou *et al.*, 2023
	Pituitary gland	AtT20 cell line	Cell cycle arrest in G0/G1 phase by decreasing Skp2 expression and increasing $p27^{kip1}$ expression	Haddad *et al.*, 2013
Lutein	Breast	BT-474, MDA-MB-453 and MDA-MB-231 cell lines	Cell cycle arrest in G0/G1 phase	Gong *et al.*, 2018
	Lung	A549 cell line and mice model	Cell cycle arrest in G0/G1 phase and increased survival in injected mice	Zhang *et al.*, 2023
Astaxanthin	Leukaemia	K562 cell line	Cell cycle arrest in G0/G1 phase via PPARγ and Keap1-Nrf2/EpRE/ARE	Zhang *et al.*, 2011
	Stomach	KATO-III and SNU-1 cell lines	Cell cycle arrest in G0/G1 phase by down-regulation of p-ERK levels	Kim *et al.*, 2016
	Breast	SKBR3 cell line	Cell cycle arrest in G0/G1 phase	Kim *et al.*, 2020
		Mice injected with 4T1 cells	Reduction in mitotic cancer cells	Shokrian *et al.*, 2024
	Colon	HCT-116 and HT-29 cell lines	Cell cycle arrest in G2/M phase by increasing CDK4 and CDK6, and decreasing $p27^{Cip1/Waf1}$, p27 and p53 expression	Liu *et al.*, 2016
Fucoxanthin	Leukaemia	MT-2, MT-4, HUT-10, ED-40515, Jurkat and K562 cell lines	Cell cycle arrest in G0/G1 phase by down-regulation of cyclins D1 and D2, and CDKs 4 and 6 levels; and up-regulating GADD45α expression	Ishikawa *et al.*, 2008

	Osteosarcoma	Saos-2, MNNG and 1438 cell lines	Cell cycle arrest in G0/G1 phase by down-regulation of cyclin E and CDKs 4 and 6 expression	Rokkaku *et al.*, 2013
	Stomach	MGC-803 cell line	Cell cycle arrest in G2/M phase by down-regulation of survivin and cyclin B1 through JAK/STAT pathway	Yu *et al.*, 2011
		BGC-823 cell line	Cell cycle arrest in G2/M phase by modulation of Mcl-1, STAT3 and p-STAT3 through JAK/STAT pathway	Yu *et al.*, 2018
		SGC-7901 cell line	Cell cycle arrest in S phase by modulation of Mcl-1, STAT3 and p-STAT3 through JAK/STAT pathway	Yu *et al.*, 2018
	Liver	HepG2 cell line	Cell cycle arrest in G0/G1 phase by increasing GADD45α, GADD153, PIM1 and CYP1A1 expression	Yoshiko *et al.*, 2007; Das *et al.*, 2008
	Prostate	DU145 cell line	Cell cycle arrest in G0/G1 phase by increasing GADD45α, GADD153 and PIM1 expression	Yoshiko *et al.*, 2007
	Skin	B16F10 cell line	Cell cycle arrest in G0/G1 phase by down-regulating cyclins D1 and D2, and CDK4 expression; and up-regulating $p15^{INK4B}$ and $p27^{Kip1}$ expression	Kim *et al.*, 2013
	Bladder	T24 cell line	Cell cycle arrest in G0/G1 phase by up-regulating p21, down-regulating CDK 2 and 4, cyclin D1 and E expression	Wang *et al.*, 2014
Capsanthin	Leukaemia	K562 cell line	Cell cycle arrest in G0/G1 phase via activation of PPARγ and Keap1-Nrf2/EpRE/ARE signalling pathways	Zhang *et al.*, 2011
	Breast	BT20, BT549, MDA-MB-468, MDA-MB-231 and MCF-10A***** cell lines	Cell cycle arrest in G0/G1 phase by up-regulating p21 only in cancer cell lines	Wu *et al.*, 2021
Crocin	Breast	Female rats with NMU-induced breast cancer	Cell cycle arrest by up-regulating of cyclin D1	Ashrafi *et al.*, 2015
	Gastric	Nude mice injected with AGS cells	Inhibition of proliferation and tumour growth	Luo *et al.*, 2021
	Colon	SW-480 cell line	Cell cycle arrest in G0/G1 phase via modulation of Akt1 and JAK2/STAT3 signalling pathways	Hosseini *et al.*, 2022

* Normal cells.

Table S2. Preclinical studies of apoptosis induction in cancer cells by carotenoids. This table summarizes the observed effects on proapoptotic capability, the specific carotenoids studied, the cancer types targeted and the experimental models used.

Carotenoid	Cancer	Model	Effect	Reference
β-carotene	Breast	MDA-MB-231 cell line	Apoptosis induction via JNK signalling	Antunes *et al.*, 2022
		MCF-7, MDA-MB-235 and MDA-MB-231 cell lines	Apoptosis induction	Gloria *et al.*, 2014
	Pituitary gland	ACTH-secreting pituitary adenoma cells	Apoptosis induction by down-regulation of Skp2	Haddad *et al.*, 2013
Lycopene	Breast	MCF-7 cell line	Apoptosis induction with PARP cleavage with ROS generation	Teodoro *et al.*, 2012; Takeshima *et al.*, 2014; Jain *et al.*, 2017; Arathi *et al.*, 2016
		SK-BR-3 and MDA-MB-468 cell lines	Apoptosis induction with PARP cleavage	Takeshima *et al.*, 2014
	Leukaemia	K562 cell line	Apoptosis induction with telomerase activity inhibition	Gharib *et al.*, 2014
	Colon	HT-29 cell line	Apoptosis induction by decreasing procaspases-8, -3 and -9, and Bcl-2; and increasing Bax levels	Teodoro *et al.*, 2012; Huang *et al.*, 2015
	Prostate	PC3 cell line	Apoptosis induction by up-regulating miR-let-7f-1 and inhibiting Akt2, with ROS generation	Li *et al.*, 2016; Arathi *et al.*, 2016
	Oesophagus	Oesophageal cancer induced F344 rats	Apoptosis induction by increasing PPARγ and caspase-3, and reducing inflammatory	Cui *et al.*, 2020

			cytokines via down-regulation NF-κB and COX-2	
	Pituitary gland	AtT20 cell line	Apoptosis induction by increasing p27 levels	Haddad *et al.*, 2013
	Uterus	HeLa cell line	Apoptosis induction with ROS generation	Arathi *et al.*, 2016
	Epidermis	A431 cell line		
	Liver	HepG2 cell line		
	Lung	A549 cell line		
Lutein	Breast	BT474, CRL-3247, MCF-7, MDA-MB-231, MDA-MB-453, MDA-MB-468, HTB-26, HTB-131 cell lines	Apoptosis induction by increasing ROS levels, p53 activation and up-regulation of HSP60	Gong *et al.*, 2018
		Primary normal human mammary epithelial cells*	Non cytotoxic effects	Sumantran *et al.*, 2000
		MCF-7 and MDA-MB-231 cell lines	Caspase-3-mediated apoptosis with intracellular ROS reduction	Kavalappa *et al.*, 2021
		Injected female BALB/c mice	Apoptosis induction by increased expression of p53 and Bax, and decreased expression of Bcl-2	Chew *et al.*, 2003
	Lung	A549, HCC827 and BEAS-2B* cell lines	Apoptosis induction by modulating PI3K-Akt pathway	Zhang *et al.*, 2018
	Stomach	AGS, MKN-74, MKN-1 and SNU-668 cell lines	Apoptosis induction by elevating ROS levels through NADPH oxidase activation	Eom *et al.*, 2023
Astaxanthin	Skin	Mouse model of metastatic melanoma with B16F10 cell line	Apoptosis induction by inhibiting Bcl-2, cyclins D1 and E, NF-κB, ERK, MEK and MMP-1 and -9, and increasing cleaved caspase-3 and -9, ATM and p21	Haung *et al.*, 2020
	Mouth	Hamster model of oral cancer	Apoptosis induction by inhibiting NF-κB and Wnt pathways and increasing Smac/Diablo and cytochrome-c cytosol levels and the cleavage of PARP.	Kavitha *et al.*, 2013
	Colon	HCT-116 cell line	Apoptosis induction by caspase-3 and PARP activation	Liu *et al.*, 2016
		HT-29 cell line	Apoptosis induction by caspase-3 and PARP activation and ROS production	Liu *et al.*, 2016; Shanmugapriya *et al.*, 2019
		LS-80 cell line	Apoptosis induction by caspase-3 activation	Hormozi *et al.*, 2019
	Breast	SKBR3 cell line	Induction of apoptosis via mutatant p53 decrease, cleavage of PARP-1 and caspase-3 and -9, activation of Bax/Bcl-2, phosphorylation of ERK1/2, JNK and p38, and reduction of ROS levels through impairment of SOD1/SOD2	Kim *et al.*, 2020
Fucoxanthin	Lung	Mouse model of lung cancer induced with benzo(a)pyrene	Induction of apoptosis by activating caspase-9 and -3 while down-regulating Bcl-2 expression	Chen *et al.*, 2019
	Leukaemia	MT-2, MT-4, HUT-10 and ED-40515(-); and Jurkat, and K562 cell lines	Induction of apoptosis by activating caspase-3, -8 and -9, and down-regulating Bcl-2, XIAP, cIAP2 and survivin	Ishikawa *et al.*, 2008
	Breast	CMT-U27 cell line	Induction of apoptosis by activating caspases-3, -7 and -8, and PARP	Jang *et al.*, 2021
	Uterus	HeLa, SiHa and CaSki cell lines	Induction of apoptosis by inhibiting the PI3K/Akt and NF-κB pathways	Jin *et al.*, 2018
	Nervous system	GBM1 cell line	Induction of apoptosis with loss of mitochondrial membrane potential and ultrastructural alterations	Lopes *et al.*, 2020
		U251 cell line	Induction of apoptosis with ROS increase and MAPKs and PI3K-Akt pathways impairments	Wu *et al.*, 2019
	Bones	Saos-2, MNNG, 143B and LM8 cell lines	Induction of apoptosis mediated by the activation of caspases-3, -8 and -9 through the down-regulation of survivin, XIAP, Bcl-2 and Bcl-xL	Rokkaku *et al.*, 2013
	Bladder	T24 cell line	Induction of apoptosis by disrupting the mortalin-p53 complex	Wang *et al.*, 2014
	Stomach	SGC-7901 and BGC-823 cell lines	Induction of apoptosis through JAK/STAT pathway	Yu *et al.*, 2018
Capsanthin	Breast	MCF-7 cell line	Induction of apoptosis with ROS generation, decreased mitochondrial membrane potential and reduced glutathione and catalase levels	Erden, 2020

		MDA-MB-231 cell line	Induction of apoptosis with ROS generation	Shanmugham *et al.*, 2022; Molnar *et al.*, 2004
	Leukaemia	K562 cell line	Induction of apoptosis by up-regulating PPARγ, p21 and Nrf2	Zhang *et al.*, 2011
**Crocin**	Retina	Y79 and WERI-RB-1 cell lines	Induction of apoptosis via MYCN down-regulation	Deng *et al.*, 2019
	Uterus	OV2008 and C13 cell lines	Induction of apoptosis by up-regulating of Bax and p53, while down-regulating Bcl-2	Mollaei *et al.*, 2017
	Breast	MDA-MB-468 and MCF10-A* cell lines	Induction of apoptosis by increasing Bax/Bcl-2 ratio and reducing HSP27, 70 and 90, only in cancer cell lines	Mostafavinia *et al.*, 2016
		MCF-7 and MDA-MB-231 cell lines	Induction of apoptosis mediated by FOXO3a	Nasimian *et al.*, 2020
**Crocetin**	Stomach	AGS and HFSF-PI3* cell lines	Induction of apoptosis by suppressing Bcl-2 and increasing Bax expression, only in cancer cell lines	Bathaie *et al.*, 2013, Hoshyar *et al.*, 2013
	Nervous system	U251, U87MG, U373 and U138 cell lines	Induction of apoptosis by supressing FASN	Colapietro *et al.*, 2020
	Oesophagus	KYSE-150 cell line	Induction of apoptosis by inhibiting PI3K/Akt, ERK1/2 and p38, and up-regulating p53 and p21	Li *et al.*, 2019
	Uterus	HeLa cell line	Induction of apoptosis via p53	Zhong *et al.*, 2011
	Breast	A549 cell line	Induction of apoptosis via p53	Zhon *et al.*, 2011
	Ovary	SKOV3 cell line	Induction of apoptosis via p53	Zhon *et al.*, 2011
	Leukaemia	HL-60 cell line and normal human polymorphonuclear* cells	Induction of apoptosis via caspases-3 and -9, and Bax/Bcl-2 ratio increment	Moradzadeh *et al.*, 2018
	Colon	HCT-116 and HT-29 cell lines	Induction of apoptosis via p53	Ray *et al.*, 2016

* Normal cells.

Table S3. Preclinical studies of the antimetastatic and antiangiogenic effect of carotenoids in cancer. This table summarizes the observed effects on metastatic capability, the specific carotenoids studied, the cancer types targeted, and the experimental models used.

Carotenoid	Cancer	Model	Effect	Reference
**β-carotene**	Stomach	H. pylori-infected AGS cell line	Inhibition of cell invasion by down-regulating MMP-10 and up-regulating catalase, with ROS reduction	Bae *et al.*, 2021
	Nervous system	SK-N-BE(2)C cell line *in vitro* and injected in immunodeficient nude mice	Inhibition of cell invasion and migration by inhibiting MMP-2, MMP-9 and HIF-1α	Kim *et al.*, 2014
	Skin	B16F10 cell line *in vitro* and C57BL/6 injected mice	Inhibition of metastasis and angiogenesis by down-regulating MMP-2 and -9, prolyl hydroxylase and lysyl oxidase and up-regulating TIMP-1 and -2	Guruvayoorappan *et al.*, 2007; Pradeep *et al.*, 2003
**Lycopene**	Head and neck	FaDu and Cal27 cell lines	Inhibition of cell invasion by down-regulating p-Akt and p-ERK	Ye *et al.*, 2016
	Colon	HT-29 cell line	Inhibition of cell invasion and migration by up-regulating of E-cadherin and down-regulating of Akt, NF-κB, pro-MMP-2 and active MMP-9.	Huang *et al.*, 2015
	Prostate	PC3 and DU145 cell lines	Inhibition of cell adhesion	Elgass *et al.*, 2014
	Spleen	SK-Hep-1 cell lines	Inhibition of cell invasion and migration by up-regulating Nm23-H1	Huang *et al.*, 2005; Hwang *et al.*, 2006
**Lutein**	Pancreas	PANC-1 cell line	Inhibition of cell invasion and migration by targeting Bcl2-associated athanogene 3 and modulating cholesterol homeostasis	Han *et al.*, 2022
	Breast	MDA-MB-157 and MCF-7 cell lines	Inhibition of cell invasion and migration by down-regulating hairy and enhancer of split 1 protein	Li *et al.*, 2018
**Astaxanthin**	Colon	HCT116 cell line and BALB/c nu/nu mice	Inhibition of metastasis by down-regulating miRNA-29a-3p and miRNA-200a	Kim *et al.*, 2019

		Wistar rats induced with dimethyl hydrazine	Inhibition of cell invasion through NF-κB, COX-2, MMPs-2/9, Akt and ERK-2	Nagendraprabhu *et al*., 2011
	Breast	T47D cell line	Inhibition of metastasis by activating mammary serine protease inhibitor, KAI1, breast cancer metastasis suppressor 1 and mitogen-activated protein kinase kinase 4	Badak *et al.,* 2021
	Stomach	H. pylori-infected AGS cell line	Inhibition of cell adhesion and migration by supressing JAK1/STAT3 activation and up-regulating AG490 and K34C	Woo *et al.,* 2023
	Nervous system	A172 cell line	Inhibition of cell invasion and migration by down-regulation MMP-2 and -9	Siangcham *et al*., 2020
Fucoxanthin	Breast	MCF-7 and 4T1 cell lines	Inhibition of metastasis by preventing adhesion and migration of CTCs	Wang *et al.,* 2022
		MDA-MB-231 cell line *in vitro* and injected in nude mice	Inhibition of lymphangiogenesis by down-regulating VEGF-C, VEGF receptor-3, NF-κB, phospho-Akt and phospho-PI3K	Wang *et al*., 2019
		human umbilical vein endothelial cells***** and CMT-U27 cell line	Inhibition of angiogenesis by up-regulating Ang2	Jang *et al.,* 2021
	Nervous system	GBM1 cells	Inhibition of cell invasion and migration	Lopes *et al*., 2020
Capsanthin			*Non-documented*	
Crocin	Stomach	Gastric cancer patients	Inhibition of EMT, migration and invasion through miR-320/KLF5/HIF-1α signalling pathway	Zhou *et al*., 2019
	Breast	4T1 cell line *in vitro* and injected BALB/c mice	Inhibition of metastasis by down-regulating VEGF and MMP-9	Farahi *et al.,* 2021
	Skin	B16F10 cell line in vitro and injected C57BL/6 mice	Inhibition of cell invasion and migration by down-regulating MMP-2 and -9, ERK-2, K-ras and VEGF	Bakshi *et al.,* 2018
	Colon	HT-29 and Caco-2 cell lines	Inhibition of angiogenesis and metastasis by blocking TNF-α/NF-κB/VEGF pathways	Bakshi *et al*., 2022
Crocetin	Stomach	NCI–N87 and Hs-746T cell lines	Inhibition of angiogenesis and metastasis through down-regulation of SHH signalling pathway	Zang *et al.,* 2021
	Breast	MDA-MB-231 cell line	Inhibition of cell invasion by down-regulating MMP-2 and -9 expression	Chryssanthi *et al.,* 2011; Chen *et al.*, 2019
	Colon	HCT-116 cell line	Inhibition of cell migration by activating the p-38 MAPK signalling pathway	Khajeh *et al.*, 2020

* Normal cells.

Table S4. Preclinical studies of less common effects of carotenoids on cancer. This table summarizes the observed effects, specific carotenoids studied, cancer types targeted and experimental models used.

Effect	Carotenoid	Cancer	Model	Reference
Autophagy	Lycopene*****	Cutaneous squamous cell carcinoma	Colo-16 cell line	Kim *et al*., 2021
	Fucoxanthin	Cervical cancer	HeLa cell line	Hou *et al*., 2013
		Nasopharyngeal carcinoma	NPC cell line	Long *et al*., 2020
	Crocin	Cervical cancer	SiHa cell line *in vitro* and in female BALB/c nude mice	Zhang *et al.,* 2020
Necroptosis	Astaxanthin	Gastric cancer	AGS cell line	IKim *et al*., 2021
Induction of cell differentiation	β-carotene	Neuroblastoma	SK-N-BE(2)C and SH-SY5Y cell lines	Kim *et al*., 2019; Lee *et al*., 2013
	Lycopene	Leukaemia	HL-60 cell line	Amir *et al*., 1999
	Crocetin	Glioma	U251 cell line	Colapietro *et al.*, 2020
Enhancement of gap junctional communication	β-carotene	Pituitary adenoma	AtT-20 cell line	Haddad *et al.,* 2013
	Lycopene			
	Fucoxanthin	Hepatoma	SK-Hep-1 cell line	Liu *et al.,* 2009
Multidrug resistance	β-carotene	Cervical cancer	KB-vin (chemoresistant) HeLaS3 (chemosensitive) cell lines	Teng *et al*., 2016
		Breast cancer	NCI-H460/MX20 (chemoresistant) and NCI-H460	Teng *et al.,* 2016

		(chemosensitive) cell lines	
Lycopene	Leukaemia	L1210 mouse cell line	Molnar *et al.*, 2004; Wang *et al.*, 2010
	Breast cancer	HTB26 cell line	Molnar *et al.*, 2004; Wang *et al.*, 2010
Lutein	Colon cancer	Colo-320-MDR (chemoresistant) and Colo-205 (chemosensitive) cell lines	Ugocsai *et al.*, 2005
	Leukaemia	L1210 mouse cell line	Molnar *et al.*, 2004
	Breast cancer	HTB26 cell line	Molnar *et al.*, 2004
	Sarcoma	S180 mouse cell line	Luan *et al.*, 2018
Fucoxanthin	Hepatoma	HepG2 cell line	Liu *et al.*, 2012; Eid *et al.*, 2020
	Colon cancer	Caco-2 cell line	Eid *et al.*, 2012
	Leukaemia	CEM/ADR5000 (chemoresistant) and CCRF-CEM (chemosensitive) cell lines	Eid *et al.*, 2012
	Breast	MCF-7/ADR cell line	Eid *et al.*, 2020
	Ovarian cancer	SKOV-3/ADR cell line	Eid *et al.*, 2020
Capsanthin	Leukaemia	L1210 mouse cell line	Molnar *et al.*, 2004; Wang *et al.*, 2010
	Breast cancer	HTB26 cell line	Molnar *et al.*, 2004; Wang *et al.*, 2010
Crocin	Gastric cancer	EPG85-257RDB cell line	Razavi *et al.*, 2020
Crocetin	Ovarian cancer	A2780-RCIS cell line	Neyshaburinezhad *et al.*, 2019; Mahdizadeh *et al.*, 2016

* In this specific model, lutein inhibits autophagy because it acts as a protumoural process.
